# Inhibitory KIR2DL2 receptor and HHV-8 in classic or endemic Kaposi sarcoma

**DOI:** 10.1007/s10238-022-00798-0

**Published:** 2022-02-15

**Authors:** Daria Bortolotti, Monica Corazza, Antonella Rotola, Dario Bencivelli, Giovanna Schiuma, Elisabetta Danese, Sabrina Rizzo, Silvia Beltrami, Roberta Rizzo, Alessandro Borghi

**Affiliations:** 1 Department of Chemical, Pharmaceutical and Agricultural Sciences, University of Ferrara, Ferrara, Italy; 2 Section of Dermatology and Infectious Diseases, Department of Medical Sciences, University of Ferrara, Ferrara, Italy

**Keywords:** Kaposi’s Sarcoma, KIR2DL2, HHV-8, Natural Killer (NK) cells

## Abstract

KIR2DL2, an inhibitory Killer cell Immunoglobulin-like Receptor (KIR), has been shown to predispose to the development of several herpesvirus-associated diseases by inhibiting the efficiency of Natural Killer (NK) cells against virus-infected cells. The aim of this observational study was to assess the prevalence of KIR2DL2 and Human Herpes Virus 8 (HHV8) in patients affected with classical and endemic Kaposi sarcoma (KS), as well as in controls. Blood samples collected from 17 Caucasian, HIV-negative, immunocompetent patients affected with classical KS (c-KS), 12 African, HIV-negative patients with endemic KS (e-KS), 83 healthy subjects and 26 psoriatic patients were processed for genotypization by PCR for two KIR alleles, such as KIR2DL2 and KIR2DL3 and analyzed for HHV-8 presence. The totality of both c-KS and e-KS patients presented HHV-8 infection, whereas HHV8 was found in 26.9% of psoriatic subjects and 19.3% of healthy subjects. KIR2DL2 was found in the 76.5% of c-KS subjects, while the receptor was found in 41.7% of the e-KS group, 34.6% of psoriatic patients and 43.4% of healthy controls (*p* < 0.0001). A significantly higher prevalence of KIR2DL2 in c-KS patients than in all the other subjects was also confirmed comparing age-matched groups. Based on these results, the inhibitory KIR2DL2 genotype appears to be a possible cofactor which increases the risk of developing c-KS in HHV8-positive, immunocompetent subjects, while it seems less relevant in e-KS pathogenesis.

## Introduction

Human Herpes Virus 8 (HHV8) was originally isolated in 1994 [13]. The infection is generally acquired during childhood mainly through saliva exchange, while blood, breast milk and semen are considered rarer sources of infection [1, 20]. Compared to the other members of the Herpes virus family, which are found almost universally in the adult population, HHV8 seroprevalence is lower and with different geographical distribution ranges. According to its prevalence, three geographical areas may be distinguished, namely (1) high endemic areas, with > 25% seroprevalence, which include many regions in Africa, (2) mid-endemic areas, with 10–25% seroprevalence (e.g., in the Mediterranean Basin) and (3) non-endemic areas, where seroprevalence is lower than 10% [35].

HHV8 infection has been associated with several malignancies, mainly of hematologic origin, including Multicentric Castleman’s Disease (MCD) and Primary Effusion Lymphomas (PEL), and post-transplant and germinotropic LPDs (GLPDs) rare lympho**-**proliferative disorders occurring prevalently in immunosuppressed subjects [13]. Moreover, a possible involvement of HHV8 in eruptive cherry angiomas (CAs) has recently been suggested [3].

The role of HHV-8 is crucial in Kaposi sarcoma (KS) development [23]. Among the KS lesions, HHV8 is predominantly found in the so-called tumor spindle cells, due to their elongated shape, which are probably endothelial cells transformed by the virus [14]. HHV8 infection reprograms, in a very polymorphic and not yet fully known way, the host’s blood endothelial cells leading to both spindle morphology acquisition and induction of a strong neo**-**angiogenic activity, which give rise to the typical KS lesions [3].

Nevertheless, HHV8 infection is not sufficient to provoke such progression, since only a small rate of HHV8-infected individuals develops KS [3]. Additional cofactors are necessary for the development of KS and the other HHV8-associated diseases.

HHV8 infection has a biphasic cycle comprising latent and lytic phases [36]. Latency is characterized by a state of quiescence in which the viral genome persists as a circular episome attached to the host chromatin, with limited expression of viral transcripts and down**-**regulated surface markers. On the contrary, the lytic phase of HHV-8 infection involves the expression of numerous genes and the production of viral particles [6, 28]. Like other herpes**-**viruses, in most cases HHV8 infection is controlled throughout life by immune surveillance. In this case, the virus remains latent within cells and clinically silent. On the other hand, since it has developed a variety of mechanisms to evade the host immune system [15, 30], HHV8 may lead to the proliferation of infected cells, up to cancer development [19]. The kinetics of virus‐associated disease progression depends on the balance of power between host immune defenses and the virus [26]. HHV8 is considered an oncogenic virus mainly in a favorable context, such as immunosuppression. In keeping with this, the risk for KS is greatly increased with concomitant human immunodeficiency virus (HIV) infection and also substantially increased in the case of chronic use of corticosteroids and immunosuppressive treatments [21].

However, it is not currently understood in detail which components of the human immune response are essential for this pathophysiological process [2, 19].

Natural Killer (NK) cells are cytotoxic leucocytes involved both in innate immune defense against viral infections and tumor cells and in the regulation of adaptive immune responses [7, 8, 12]. NK cells are key effectors of the immune system due to their human leukocyte antigen (HLA)-restricted cytotoxic activity [7]. Killer cell immunoglobulin-like receptors (KIR), which are highly polymorphic members of the immunoglobulin superfamily, regulate human NK cell development and functions. A number of KIRs located in different haplotypes have been identified, with either inhibitory or activating functions on NK cells. Thus, KIR gene diversity concurs in determining NK cells**’** efficiency against transformed or virus-infected cells and, accordingly, the susceptibility of subjects to symptomatic herpesvirus infection [18, 25]. Specific inhibitory KIRs present on NK cells have been shown to impair the anti-herpetic immune response, predisposing to susceptibility to herpesvirus infection. In particular, subjects expressing KIR2DL2 on NK cells fail to control herpes simplex virus 1 (HSV-1), Epstein-Barr virus (EBV), Human Herpes Virus-6 (HHV-6) and HHV8 infections [9, 10, 31, 33]. This specific KIR allotype appears to predispose to the development of herpesvirus-associated diseases as well, such as type 2 diabetes, Alzheimer disease, Multiple Sclerosis and eruptive cherry angiomas [4, 9, 10, 31, 33].

On the base of these data, a potential role of KIR genotype in KS pathogenesis as well might be supposed, even though the actual scenario is not clearly defined. Indeed, the combination of the activating haplotype KIR3DS1 and HLA-B Bw4-80I has been found to increase the risk of classic KS in a cohort of HHV8-positive and HIV-negative patients, [21] but not in HIV-positive subject [29]. In a cohort of Italian classic KS patients, the KIR2DS1 with its HLA-C2 ligand as well as the activating haplotypes KIR3DS1 and KIR2DS1 were statistically more frequent among classic KS cases than controls [22].

These previous findings support the hypothesis that KIR gene polymorphisms can, to some extent, account for the complex interactions between genetic background, HHV8 infection and the immune system, as cofactors in KS development.

Starting from this assumption, the main aims of the present study were to assess the prevalence of the inhibitory KIR2DL2 receptor in HIV-negative patients affected with classical KS and to compare it to that of African patients with endemic KS, psoriatic patients and healthy controls. This assessment could provide some insights into inhibitory KIR genotype involvement in the physiopathological process of KS.

## Materials and methods

### Study populations

In the present mono-centric, observational study we included all adult (≥ 18 yrs.), Caucasian, HIV-negative subjects affected with histologically proven classic KS consecutively referring to our outpatient Dermatology clinics over a 6-month period. Patients were excluded in the presence of the following: (1) lack of histological confirmation, (2) presence of ethnic, clinical, laboratory or history criteria suggesting varieties of KS other than the classical one, (3) chronic immunosuppressive treatments for any health condition, (4) refusal to undergo blood sampling or inclusion in the study.

The following three different control populations were recruited: (1) healthy donor subjects, (2) psoriatic patients and (3) HIV-negative African patients affected with endemic KS. Both healthy donors and psoriatic patients, whose blood samples were stored in the archive of the laboratory of the University of Ferrara, were contacted for their agreement to use part of their blood samples for the specific purposes of this study. We established a priori the inclusion of healthy controls and psoriatic patients in order to have a gender distribution comparable to that of the enrolled KS patients. No age-based selections were made. Only the blood samples belonging to subjects who gave their consent were used. Healthy controls and psoriatic patients did not have concurrent documented infectious diseases. No further exclusion criteria were applied. Endemic KS skin lesions from Ugandan patients, stored in the archive of the laboratory of the University of Ferrara since their use in a previous study [24], were the biological source for this control population.

This was a spontaneous survey, with no funding from external sources. The principles outlined in the Helsinki Declaration of 1975, as revised in 1983, have been followed for all the patients included. The study was approved by the University-Hospital of Ferrara institutional review board (project identification code 162/2020/Oss/AOUFe). Both patients and controls, with the sole exception of patients with endemic KS, provided their written informed consent.

### KIR2DL2 genotypization and HHV-8 search

Blood samples collected from the four study populations were processed for genomic DNA extraction using the QIAamp DNA blood Mini Kit (QIAGEN, Hilden, Germany) following the manufacturer’s protocol [32].

KIR alleles (KIR2DL2 and KIR2DL3) were genotyped by PCR by specific primers pairs [17] reported in Table 1 and visualized on 1% agarose gel, as previously reported [34].

**Table 1:** KIR2DL2/KIR2DL3 and HHV-8 ORF50 specific primer sets and amplified fragments length

Gene	Primers	Sequence	Fragment legth (bp)
KIR2DL2	K2DL2f	CCA TGA TGG GGT CTC CAA A	1800 bp
	K2DL2r	GCC CTG CAG AGA ACC TAC A	
KIR2DL3	K2DL3f	CCT TCA TCG CTG GTG CTG	798 bp
	K2DL3r	CAG GAG ACA ACT TTG GAT CA	
ORF50	8–50-1	TTG GTG CGC TAT GTG GTC TG	420 bp
	8–50-2	GGA AGG TAG ACC GGT TGG AA	
ORF50 nested	8–50-3	TAT TCG GAT CCT CAC GGA GA	227 bp
	8–50-4	CGG TAT CGT ACG TGT TGT AG	

HHV-8 genome was detected by PCR**,** specific for ORF50 gene, followed by nested PCR (Table 1), as previously described [10].

### Statistical analysis

Results were evaluated for statistical significance by GraphPad Prism v.9 software. Frequency difference was analyzed by χ^2^-test, while difference between mean values was determined by Student-t test. *P*-values < 0.01 were considered statistically significant.

## Results

### Study populations

All the 17 eligible patients affected with classical KS screened for entry into the study were included. Based on the inclusion and exclusion criteria, biological samples from 83 healthy subjects, 26 psoriatic patients and 12 African patients affected with endemic KS were included. Demographic characteristics of the four study populations are shown in Table 2.

**Table 2:** Demographic characteristics of the study populations

Demographic features	c-KS (*n* = 17)	e-KS (*n* = 12)	Psoriatic patients (*n* = 26)	Healthy controls (*n* = 83)
Sex, n. (%)				
Males	16 (94.1%)	10 (83%)	24 (92.3%)	78 (93.9%)
Females	1 (5.9%)	2 (17%)	6 (7.7%)	5 (6.1%)
Age, mean ± SD [range]	77.3 ± 11 [47–95]	32 ± 9.8 [22–48]	53.7 ± 7.4 [28–81]	55.1 ± 10.6 [26–96]

c-KS, HIV-negative classic Kaposi Sarcoma patients; e-KS, HIV-negative endemic Kaposi Sarcoma patients; SD, standard deviation

### HHV8 seroprevalence

**Fig. 1:**
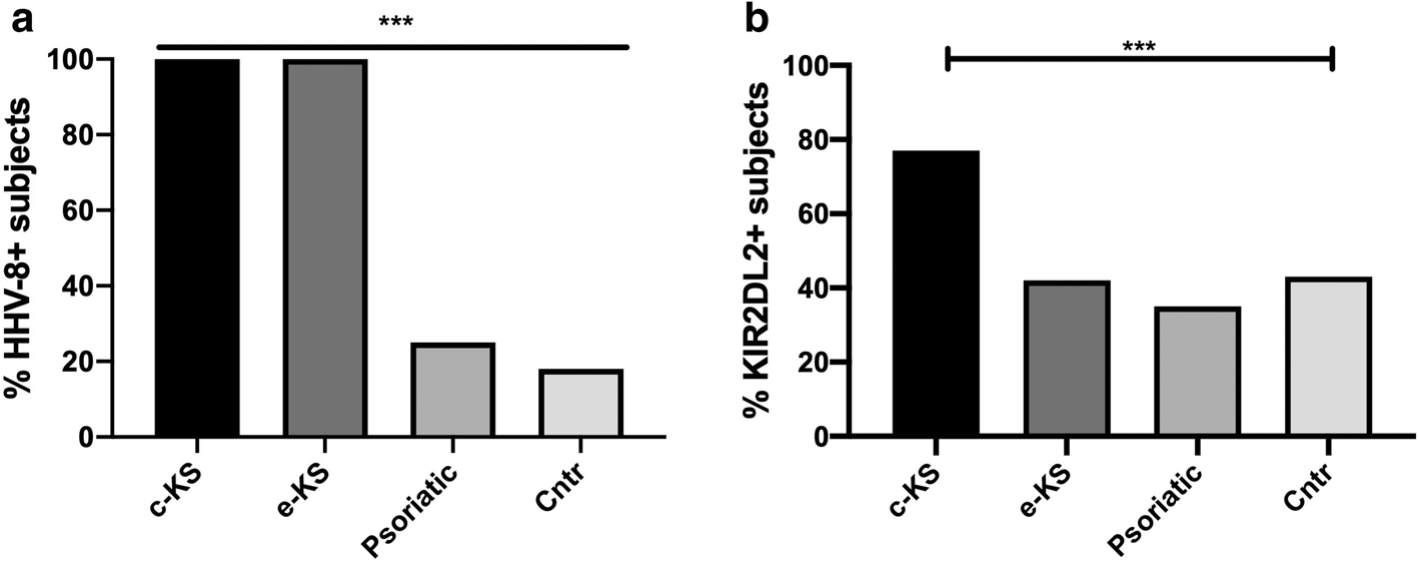
Assessment of HHV-8 **a** and KIR2DL2 **b** frequencies in HIV-negative classic KS **c**-KS, endemic KS **e**-KS, psoriatic and control (cntr) groups. *** *p* < 0.0001

HIV-negative, Caucasian classic KS (c-KS) and African endemic KS (e-KS) patients were characterized for the presence of HHV-8 infection and compared to psoriatic and healthy groups. As shown in Fig. 1a, we found that the totality of both c-KS and e-KS patients presented HHV-8 infection. On the contrary HHV8 was found in 5 (26.9%) and in 16 (19.3%) of the psoriatic and healthy subjects, respectively (*p* < 0.0001, χ^2^-test).

### KIR2DL2 presence was associated with HHV-8 infection in classic KS, but not in endemic KS patients

The same groups were also compared considering KIR2DL2 frequency (Fig. 1b). We considered as KIR2DL2-positive subjects (KIR2 +) those who presented either KIR2DL2 homozygous or KIR2DL2/KIR2DL3 heterozygous genotypes. KIR2DL3 homozygous subjects were considered as KIR2DL2-negative (KIR2-). We found the highest KIR2DL2 frequency in the c-KS group (13/17, 76.5%), while the presence of this receptor was significantly lower among the other three groups analyzed (5 out of 12 e-KS patients, i.e., 41.7, 9/26, 34.6%, psoriatic patients, and 36/83, 43.4%, healthy controls; *p* < 0.0001, χ^2^-test; these rates were all comparable to each other).

**Fig. 2:**
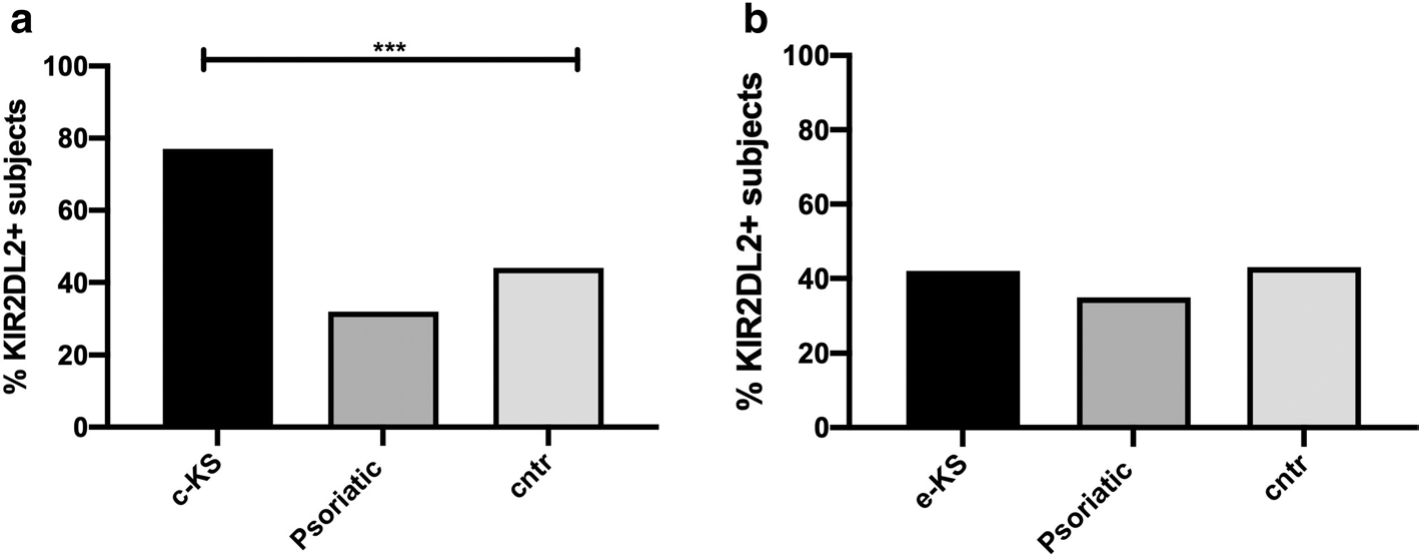
Evaluation of KIR2DL2 frequencies in HIV-negative classic KS (c-KS) **a**, endemic KS (e-KS) **b** compared with aged-matched psoriatic and control (cntr) groups. *** *p* < 0.0001

Given the significant difference between c-KS and e-KS groups’ mean age (77.3 *vs* 32 years, respectively; *p* < 0,0001, Student-t test), in order to verify the reliability of these results regardless of age, we analyzed KIR2DL2 frequency dividing both the psoriatic and healthy groups accordingly with the mean ages of c-KS and e-KS patients (Table 3). As shown in Fig. 2, the age-matched comparison confirmed the previous results, reporting a significantly higher frequency of KIR2DL2 in the c-KS group (Fig. 2a, p < 0,0001, χ^2^-test), but not in the e-KS group (Fig. 2b), compared to controls.

**Table 3:** Demographical characteristics of classic (c-KS) and endemic Kaposi sarcoma (e-KS) age-matched psoriatic and control groups

	c-KS age-matched		e-KS age-matched	
Demographic features	Psoriatic patients (*n* = 16)	Healthy controls (*n* = 50)	Psoriatic patients (*n* = 10)	Healthy controls (*n* = 33)
Sex, n. (%)				
males	15 (93.8%)	48 (96%)	9 (90%)	30 (91%)
females	1 (6.2%)	2 (4%)	1 (10%)	3 (9%)
Age, mean ± SD [range]	69.6 ± 8.4 [54–81]	73.6 ± 10.6 [54–96]	37.8 ± 6.4 [28–48]	36.5 ± 10.5 [26–49]

c-KS, HIV-negative classic Kaposi Sarcoma patients; e-KS, HIV-negative endemic Kaposi Sarcoma patients; SD, standard deviation

## Discussion

In the present study we assessed the prevalence of a specific inhibitory KIR receptor, namely KIR2DL2, among KS patients in order to evaluate its possible role on HHV8 seroprevalence and KS development. In particular, we were interested in assessing its potential involvement in two varieties of KS, i.e., classic and endemic KS, in comparison with controls. We chose to investigate KIR2DL2, among others, since this receptor has been found, even by us, to facilitate herpes**-**virus infections, including those from HHV-8 [4, 9, 10, 31, 33]. The presence of KIR2DL2 appears to predispose to development of herpesvirus associated diseases as well. It has been supposed that its inhibitory function on NK cells may create favorable immunological conditions for HHV8 to carry out its functions, including angioproliferative and oncogenic activity, in infected individuals. Based on these observations, the present study aimed at analyzing the possible interplay among KIR2DL2, HHV8 infection and classic and endemic KS. In this exploratory phase of the study, we did not include patients affected with epidemic KS, since, in our opinion, in this specific subset the immunodeficiency induced by HIV could diminish, or overwhelm or hide, the relevance of KIR arrangement in the physiopathological cascade of KS.

Our first finding was that the totality of subjects were positive for HHV8 in both c-KS and e-KS cohort, as expected. This confirms the well-recognized etiologic role of HHV8 in KS. On the other hand, HHV8 seroprevalence among psoriatic and healthy subjects was fully in line with its prevalence in the general population resident in a mid-endemic area, like the province of Ferrara, northern Italy [15, 33].

With reference to the main aim of this study, we found that KIR2DL2 prevalence was significantly higher in c-KS patients than in all the other study groups, including e-KS patients (Fig. 1). A higher prevalence of KIR2DL2 in c-KS patients was confirmed also comparing age-matched groups (Fig. 2). This finding suggests that KIR2DL2 is more relevant for the development of KS in Mediterranean subjects than for HHV8 infection in general. In fact, e-KS patients did not show a different prevalence of this KIR genotype when compared with controls, despite a significantly higher HHV8 seroprevalence. This is in line with previous results, which showed that in KS patients HHV8 seroprevalence had no significant association with any individual KIR gene or HLA allele [21, 22]. On the other hand, more than 76% of the c-KS patients were positive for KIR2DL2. Thus it can be supposed that in Caucasian, HIV-negative, nonimmune-compromised, HHV8-positive subjects, this KIR genotype may be a determinant for the risk of developing KS. In the aforesaid subjects, without further known predisposing factors, the following setting can be hypothesized. A particular KIR genotype, such as KIR2DL2, by binding HLA class I molecules, inhibits NK cell-mediated destruction of virus-infected and transformed tumor cells. This probably leads to an uncontrolled HHV8 activity, which may predispose to malignant transformation of infected cells and tumor progression, which underlie KS. On the other hand, in e-KS subjects, further cofactors seem to have a major relevance in KS pathogenesis than KIR2DL2. However, possible cofactors determining the high prevalence of e-KS in endemic African areas remain unknown [16].

Our results may appear in contrast with previous findings [21, 22]. In fact, two previous studies found a prevalent presence of activating KIR/HLA receptor/ligand combinations in c-KS. The authors of those studies hypothesized that the inflammation triggered by activated NK cells could be a key component of the oncogenesis, contributing to the proliferation and survival of tumor cells. It cannot be excluded that the different geographical origin of the c-KS patients included in these studies may, at least in part, account for this difference.

The study results should be viewed in light of some limitations. First of all, the small sample size of the study might have reduced the statistical power of these findings. Larger studies including a higher number of patients are needed to confirm our results. Furthermore, the HLA counterpart of the KIR2DL2 receptor was not typed. As some of us have shown in previous studies conducted under different clinical settings, this point would be important in order to know the proportion of patients with a possible activation of KIR-HLA complex [9]. The c-KS patients belong to a mid-endemic area for HHV8 seroprevalence. Thus, they may not be fully representative for patients from different geographical sites and a possible selection bias cannot be excluded. The e-KS skin samples originated from a historical population of HIV-negative Ugandan subjects of whom some relevant information had not been collected at the time, such as co-morbidities or further co-infections.

In conclusion, our study assessed for the first time the prevalence of the inhibitory KIR allotype KIR2DL2 in HIV-negative patients affected with classical KS, in comparison with African subjects affected with e-KS and controls. Based on our results, KIR2DL2 did not seem to influence the HHV8 seroprevalence in KS subjects, while it was significantly more present in c-KS patients than in the other groups. A likely impairment of NK-mediated immunity, due to the inhibitory KIR2DL2 genotype, may be a cofactor that increases the risk of developing c-KS in HHV8-positive subjects. This specific genotype may be one of the predisposing factors that concur on the development of KS in a part of subjects positive for HHV8, in the absence of further recognized risk factors. Our results also highlighted the different relevance of KIR2DL2 on the outcome of HHV8 infection in HIV-negative Caucasian and African endemic KS populations.

## References

[1] Becerril S, Corchado-Cobos R, García-Sancha N (2021). Viruses and skin cancer. Int J Mol Sci.

[2] Blumenthal MJ, Cornejo Castro EM, Whitby D (2021). Evidence for altered host genetic factors in KSHV infection and KSHV -related disease development. Rev Med Virol.

[3] Borghi A, Benedetti S, Corazza M (2013). Detection of human herpesvirus 8 sequences in cutaneous cherry angiomas. Arch Dermatol Res.

[4] Borghi A, D’Accolti M, Rizzo R (2016). High prevalence of specific KIR types in patients with HHV-8 positive cutaneous vascular lesions: a possible predisposing factor?. Arch Dermatol Res.

[5] Broussard G, Damania B (2020). KSHV: immune modulation and immunotherapy. Front Immunol.

[6] Cai Q, Verma SC, Lu J, Robertson ES, Maramorosch K, Shatkin A, Murphy F (2010). Molecular biology of Kaposi’s sarcoma-associated herpesvirus and related oncogenesis. Advances in virus research.

[7] Caligiuri MA (2008). Human natural killer cells. Blood.

[8] Carroll MC, Prodeus AP (1998). Linkages of innate and adaptive immunity. Curr Opin Immunol.

[9] Caselli E, Rizzo R, Ingianni A (2014). High prevalence of HHV8 infection and specific killer cell immunoglobulin-like receptors allotypes in Sardinian patients with type 2 diabetes mellitus: HHV8 and KIR prevalence in Type 2 diabetes. J Med Virol.

[10] Caselli E, Soffritti I, D’Accolti M (2019). HHV-6A infection and systemic sclerosis: clues of a possible association. Microorganisms.

[11] Cattani P, Cerimele F, Porta D (2003). Age-specific seroprevalence of Human Herpesvirus 8 in Mediterranean regions. Clin Microbiol Infect.

[12] Cerwenka A, Lanier LL (2001). Natural killer cells, viruses and cancer. Nat Rev Immunol.

[13] Chang Y, Cesarman E, Pessin M (1994). Identification of herpesvirus-like DNA sequences in AIDS-associated Kaposi’s sarcoma. Science.

[14] Cousins E, Nicholas J, Jeang K, Chang MH (2014). Molecular biology of human herpesvirus 8: novel functions and virus-host interactions implicated in viral pathogenesis and replication. Viruses and human cancer.

[15] Dal Maso L, Polesel J, Ascoli V (2005). Classic Kaposi’s sarcoma in Italy, 1985–1998. Br J Cancer.

[16] Dedicoat M, Newton R (2003). Review of the distribution of Kaposi’s sarcoma-associated herpesvirus (KSHV) in Africa in relation to the incidence of Kaposi’s sarcoma. Br J Cancer.

[17] Du Z, Gjertson DW, Reed EF (2006). Receptor-ligand analyses define minimal killer cell Ig-like receptor (KIR) in humans. Immunogenetics.

[18] Estefanía E, Gómez-Lozano N, Portero F (2007). Influence of KIR gene diversity on the course of HSV-1 infection: resistance to the disease is associated with the absence of KIR2DL2 and KIR2DS2. Tissue Antigens.

[19] Fang Q, Liu Z, Zhang T (2019). Human leukocyte antigen polymorphisms and Kaposi’s sarcoma-associated herpesvirus infection outcomes: a call for deeper exploration. J Med Virol.

[20] Ganem D (2010). KSHV and the pathogenesis of Kaposi sarcoma: listening to human biology and medicine. J Clin Invest.

[21] Goedert JJ, Martin MP, Vitale F (2016). Risk of classic Kaposi sarcoma with combinations of killer immunoglobulin-like receptor and human leukocyte antigen loci: a population-based case-control study. J Infect Dis.

[22] Guerini FR, Mancuso R, Agostini S (2012). Activating KIR/HLA complexes in classic Kaposi’s Sarcoma. Infect Agent Cancer.

[23] Karen M, Chang Y (2000). Kaposi's Sarcoma. N Engl J Med.

[24] Monini P, Rotola A, de Lellis L (1996). Latent BK virus infection and Kaposi's sarcoma pathogenesis. Human Cancer.

[25] Moraru M, Cisneros E, Gómez-Lozano N (2012). Host genetic factors in susceptibility to herpes simplex type 1 virus infection: contribution of polymorphic genes at the interface of innate and adaptive immunity. J Immunol.

[26] Münz C (2020). Probing reconstituted human immune systems in mice with oncogenic γ-Herpesvirus infections. Front Immunol.

[27] Oksenhendler E, Boutboul D, Galicier L (2019). Kaposi’s Sarcoma-associated Herpesvirus/Human Herpesvirus 8 associated lymphoproliferative disorders. Blood.

[28] Pardamean CI, Wu T-T (2021). Inhibition of host gene expression by KSHV: sabotaging mRNA stability and nuclear export. Front Cell Infect Microbiol.

[29] Qi Y, Martin MP, Gao X (2006). KIR/HLA pleiotropism: protection against both HIV and opportunistic infections. PLoS Pathog.

[30] Radu O, Pantanowitz L (2013). Kaposi Sarcoma. Arch Pathol Lab Med.

[31] Rizzo R, Bortolotti D, Fainardi E (2016). KIR2DL2 inhibitory pathway enhances Th17 cytokine secretion by NK cells in response to herpesvirus infection in multiple sclerosis patients. J Neuroimmunol.

[32] Rizzo R, Bortolotti D, Gentili V (2019). KIR2DS2/KIR2DL2/HLA-C1 haplotype is associated with Alzheimer’s disease: implication for the role of herpesvirus infections. J Alzheimers Dis.

[33] Rizzo R, Gentili V, Casetta I (2012). Altered natural killer cells response to herpes virus infection in multiple sclerosis involves KIR2DL2 expression. J Neuroimmunol.

[34] Rizzo R, Gentili V, Rotola A (2014). Implication of HLA-C and KIR alleles in human papillomavirus infection and associated cervical lesions. Viral Immunol.

[35] Tornesello ML, Biryahwaho B, Downing R (2010). Human herpesvirus type 8 variants circulating in Europe, Africa and North America in classic, endemic and epidemic Kaposi’s sarcoma lesions during pre-AIDS and AIDS era. Virology.

[36] Ye F, Lei X, Gao S-J (2011). Mechanisms of Kaposi’s sarcoma-associated herpesvirus latency and reactivation. Adv Virol.

